# Quality of life among caregivers of people with end-stage kidney disease managed with dialysis or comprehensive conservative care

**DOI:** 10.1186/s12882-020-01830-9

**Published:** 2020-05-04

**Authors:** Karan K. Shah, Fliss E. M. Murtagh, Kevin McGeechan, Susan M. Crail, Aine Burns, Rachael L. Morton

**Affiliations:** 1 National Health and Medical Research Council (NHMRC) Clinical Trials Centre, The University of Sydney, 92-94 Parramatta Road, Camperdown, NSW 2050 Australia; 2 Wolfson Palliative Care Research Centre, Hull York Medical School, University of Hull, Kingston upon Hull, UK; 3 School of Public Health, The University of Sydney, Sydney, NSW Australia; 4 Royal Adelaide Hospital, Adelaide, SA Australia; 5 Royal Free Hospital, London NHS Foundation Trust, London, UK

**Keywords:** Informal caregivers, Chronic renal insufficiency, Quality of life, Renal Dialysis, Conservative care

## Abstract

**Background:**

To measure health-related and care-related quality of life among informal caregivers of older people with end-stage kidney disease (ESKD), and to determine the association between caregiver quality of life and care recipient’s treatment type.

**Methods:**

A prospective cross-sectional study was conducted. Three renal units in the UK and Australia were included. Informal caregivers of people aged ≥75 years with ESKD managed with dialysis or comprehensive conservative non-dialytic care (estimated glomerular filtration (eGFR) ≤10 mL/min/1.73m^2^) participated. Health-related quality of life (HRQoL) was assessed using Short-Form six dimensions (SF-6D, 0–1 scale) and care-related quality of life was assessed using the Carer Experience Scale (CES, 0–100 scale). Linear regression assessed associations between care-recipient treatment type, caregiver characteristics and the SF-6D utility index and CES scores.

**Results:**

Of 63 caregivers, 49 (78%) were from Australia, 26 (41%) cared for an older person managed with dialysis, and 37 (59%) cared for an older person managed with comprehensive conservative care. Overall, 73% were females, and the median age of the entire cohort was 76 years [IQR 68–81]. When adjusted for caregiver sociodemographic characteristics, caregivers reported significantly worse carer experience (CES score 15.73, 95% CI 5.78 to 25.68) for those managing an older person on dialysis compared with conservative care. However, no significant difference observed for carer HRQoL (SF-6D utility index − 0.08, 95% CI − 0.18 to 0.01) for those managing an older person on dialysis compared with conservative care.

**Conclusions:**

Our data suggest informal caregivers of older people on dialysis have significantly worse care-related quality of life (and therefore greater need for support) than those managed with comprehensive conservative care. It is important to consider the impact on caregivers’ quality of life when considering treatment choices for their care recipients.

## Background

Older people with end-stage kidney disease (ESKD) managed with dialysis have ageing-related health and social care needs, and a high likelihood of developing frailty syndrome within a few months of starting dialysis [1–4]. Informal care of older people or those with chronic disease is largely provided by their family members and close friends [5]. Informal care tasks include assistance with activities of daily living, support with mobility, transportation, social support and personal care (e.g. washing and dressing). Although generally unpaid, some caregivers may receive a nominal payment or state benefits [6].

Observational data suggest dialysis may not extend life in the very old, or those with multiple comorbidities and poor physical function [1, 7–9] and it is not surprising a high proportion of older people choose comprehensive conservative care (i.e. no dialysis but active supportive care) in health systems where this option is actively discussed, (Canada, the UK and Australia) [1, 10, 11]. With the rapidly growing number of elderly people with ESKD and those with comorbid conditions receiving kidney replacement therapy, the burden on informal caregivers (i.e. close friends or family) to provide care and support has increased [12].

Caregiving demands in managing dialysis has proved to be taxing on the physical, social and emotional health of informal caregivers [12, 13]. Previous research shows that caregivers may experience depression, anxiety, fatigue, social isolation, relationship strains, financial difficulties and stress due to the added responsibility of managing their care recipient’s treatment, dietary requirements, clinic appointments and psychosocial issues [13–21]. However, robust comparative evidence on the health-related and care-related quality of life of informal caregivers of older people managed with dialysis or comprehensive conservative care is limited. This is important because family members are actively encouraged to participate in ESKD modality decision-making – and they need to be informed. Furthermore, many patients also consider the potential impact on their close persons in making decisions about treatment [22, 23].

Previous economic evaluations of healthcare interventions for caregivers have limited the assessment of health benefit to solely health-related quality of life (HRQoL) [5, 24–28]. These benefits were typically measured in utility weights, also called health state preferences that are combined with survival time to obtain quality-adjusted life-years (QALYs). However, it should be noted that QALYs were not developed to capture caregivers quality of life and previous research suggests that they may be insensitive to psychological and broader effects of caregiving [5, 29–31]. Other HRQoL measures included specific “sum score” measures of carer quality of life such as the Carer Strain Index (not preference-based) and Sense of Competence Questionnaire (validated for informal caregivers of patients with diagnosed dementia and stroke) [5, 32, 33].

The Carer Experience Scale (CES) was constructed to record the caring experience, calculate caregiver quality of life, and could be used in the assessment of interventions targeted towards caregivers [5, 34]. The CES produces a single score reflecting the overall effect of caregiving and is preference weighted (i.e. constitutes the value or desirability of caregivers of older people in the UK) that quantifies the relative importance of the caregiving domains, where some caring tasks might be perceived more burdensome than others [5].

We aimed to assess and compare the health-related quality of life (the gold standard required for economic evaluations) and care-related quality of life among informal caregivers of older people with ESKD, managed with dialysis or comprehensive conservative care; and explore associations between the caregiver’s quality of life and care recipient’s treatment type.

## Methods

### Study design

We conducted a multicentre prospective cross-sectional study in two countries of informal caregivers of older people with ESKD treated with dialysis or comprehensive conservative care, between 2014 and 2017. The study was performed in accordance with the Australian National Statement on Ethical Conduct in Human Research (2007), and National Research Ethics guidance in the UK. Each renal unit participating in the study obtained the approval of the Institutional Health Research Ethics Committee to conduct the study. The study was reported using Strengthening the Reporting of Observational Studies in Epidemiology (STROBE) guidelines for observational studies (Additional file item 1) [35].

### Setting and participants

The study was undertaken at three tertiary renal units in the UK and Australia [36]. Patients aged ≥75 years with ESKD, managed with dialysis (facility haemodialysis, home haemodialysis, or peritoneal dialysis); or managed with comprehensive conservative, non-dialytic care and with an estimated glomerular filtration (eGFR) ≤10 ml/min/1.73m^2^, were asked to nominate one or more of their informal caregivers (i.e. partners, siblings, relatives, or close friends) to participate. Caregivers present at appointments were invited to participate. In addition, patients who participated took an information sheet home for their caregivers and if interested were invited to participate. One renal unit mailed out surveys if the patient thought their caregiver may be interested. Interested caregivers were asked to sign the consent form and provided with the survey booklet consisting of SF-12 (Additional file item 2) and CES questionnaire (Additional file item 3) along with questions assessing their sociodemographic characteristics (Additional file item 4).

### Variables

The main outcome variables were the caregiver SF-6D utility (a generic preference-based single measure of HRQoL) on a 0 to 1 scale (death to full health, where a higher utility scores indicate higher HRQoL), and CES scores (measure of care-related quality of life) on a 0 to 100 scale (‘worst caring experience’ to ‘best experience’, where higher scores indicate higher care-related quality of life).

The SF-12 responses were transformed into HRQoL weights, known as utilities, using a published SF-6D algorithm [37]. The SF-6D encompasses six multi-level dimensions: *“physical functioning, role limitations, social functioning, pain, mental health, and vitality”*. The SF-6D utilities were calculated using UK population values [37–39].

The CES preference-based questionnaire measures care-related quality of life and consists of six dimensions of caregiving: *“* [1] *activities outside caring* [2]*; support from family and friends (social support)* [3]*; assistance from organizations and the government (institutional support)* [4]*; fulfilment from caring* [5]*; control over caring and* [6] *getting-on with the care recipient”* [34, 40]. It has three-level response options, representing three levels of caregiver experience. The CES produces a single score reflecting the overall effect of caregiving and is preference weighted (using UK population tariffs) [5].

### Data sources/measurement

Data were collected using a pen and paper-based survey at a single time point (cross-sectional).

### Study size

The study did not require a specific power calculation as the sample size was determined by the requirements of the original ICECAP-O study [36] assessing patient quality of life and wellbeing. Multiple caregivers attached to a single older person with ESKD were permitted to participate, given the known difficulties in identifying and recruiting informal caregivers to research studies.

### Quantitative variables

The SF-6D utilities, CES scores, and caregivers’ age were treated as continuous, while caregivers’ sex, country (UK, Australia), care recipient treatment type (dialysis, conservative care), education level (some high school or lower, completed high school or higher), private health insurance (yes, no), care recipient length of kidney disease (less than 1 year, 1–2 years, more than 2 years), length of caring (0–2 years, more than 2 years), type of relationship to the care recipient (spouse/partner, child, sibling, other) were analysed as categorical variables. Age was additionally dichotomised (less than or equal to, versus greater than the median age [76 years]).

### Statistical methods

Descriptive statistics were used to assess proportions and mean values of SF-6D utilities, and the CES score. Chi-square test was used to determine the differences in proportion of caregiver characteristics. Hypothesis testing with a two-tailed Student’s t-test was used to determine associations in mean values of SF-6D utilities, and CES score by care recipient treatment type and caregiver socio-demographic characteristics. One-way Analysis of Variance (ANOVA) was used to determine differences in the means for ‘type of relationship with the care recipient’ variable with three independent categories. Generalised linear regression with multivariable models (GLM) was undertaken to determine the association between care recipient treatment type on SF-6D utilities, and CES scores, adjusted for caregiver characteristics. Age, sex, country, education, private health insurance, care recipient treatment type, care recipient‘s duration of kidney disease, length of caring, and type of relationship with care recipient were included as covariates on the basis of a priori knowledge of their associations with the HRQoL and care-related quality of life.

Complete case analysis was performed for all outcomes. All analyses were undertaken with SAS Version 9.4 (SAS Institute, Cary, NC). A *p*-value of < 0.05 was considered statistically significant.

## Results

A total of 63 caregivers were enrolled (Additional file figure 1), 49 (78%) were from Australia, 26 (41%) cared for an older person managed with dialysis, and 37 (59%) cared for an older person managed with comprehensive conservative care. Overall, 73% were females, and the median age of the entire cohort was 76 years [IQR 68–81]. Overall, the conservative care group had higher rate of children performing the caring (*p* = 0.01); Australian participants (*p* = 0.05); and care recipients with kidney disease for more than 2 years (*p* = 0.006) compared with the dialysis group. Caregiver characteristics are shown in Table 1.

**Table 1:** Caregiver characteristics according to care recipient treatment group

Caring Context	Dialysis ***n*** = 26 n (%)	Conservative Care ***n*** = 37 n (%)	Total ***n*** = 63 n (%)	***P*** value
**Caregivers of care recipient on dialysis**				–
Facility Hemodialysis	13 (50%)	–	13 (21%)	
Home Hemodialysis	1 (4%)	–	1 (2%)	
Peritoneal Dialysis	12 (46%)	–	12 (19%)	
**Median age** (y)	76 [70–79]	76 [68–82]	76 [68–81]	
**Age group**				0.62
*≤* 76 years	15 (58%)	19 (51%)	34 (54%)	
> 76 years	11 (42%)	18 (49%)	29 (46%)	
**Gender**				0.56
Males	6 (23%)	11 (30%)	17 (27%)	
Females	20 (77%)	26 (70%)	46 (73%)	
**Health System**				0.05*
United Kingdom	9 (35%)	5 (14%)	14 (22%)	
Australia	17 (65%)	32 (86%)	49 (78%)	
**Education**				0.68
Primary school	5 (19%)	10 (27%)	15 (24%)	
Some high school	7 (27%)	11 (30%)	18 (28%)	
Completed high school	6 (23%)	7 (19%)	13 (21%)	
Completed diploma	3 (12%)	6 (16%)	9 (14%)	
Completed university degree	5 (19%)	3 (8%)	8 (13%)	
**Private health insurance**				0.90
Yes	17 (65%)	25 (67%)	42 (67%)	
No	8 (31%)	11 (30%)	19 (30%)	
Unknown	1 (4%)	1 (3%)	2 (3%)	
**Care recipient length of kidney disease**				0.006*
< 1 year	2 (8%)	–	2 (3%)	
1–2 years	5 (19%)	–	5 (8%)	
> 2 years	19 (73%)	34 (100%)	53 (88%)	
**Length of care**				0.23
0–2 years	7 (28%)	5 (15%)	12 (21%)	
> 2 years	18 (72%)	28 (85%)	46 (79%)	
**Type of relationship**				0.01*
Spouse/Partner	19 (73%)	20 (63%)	39 (67%)	
Child	–	9 (28%)	9 (16%)	
Sibling	1 (4%)	–	1 (1%)	
Other	6 (23%)	3 (9%)	9 (16%)	

* *p* < 0.05, statistical significance

### Health-related quality of life (SF-6D utility index)

Of 63 informal caregivers, the mean utility for 58 with complete data was 0.74 (SD 0.13). The mean utility of caregivers of patients managed with dialysis was 0.70 (SD 0.13), and 0.77 (SD 0.12) for those caring for conservative care patients (Additional file Table 1). The “vitality” domain reported the highest average score and was responsible for the highest decrement in utilities for caregivers of the patients managed with dialysis or conservative care (Additional file Table 2).

When adjusted for other variables, there was no significant difference by care recipient treatment type (Table 2).

**Table 2:** Adjusted differences in SF-6D utility according to caregiver sociodemographic characteristics and care recipient treatment group

	Differences	95% Lower CI	95% Upper CI	*p* value
**Age (Y)**	−0.002	−0.007	0.002	0.34
Gender	−0.04	−0.13	0.05	0.36
**Care recipient treatment**	−0.08	−0.18	0.01	0.09
**Health System**	−0.05	− 0.15	0.06	0.36
**Education**	0.04	−0.06	0.13	0.44
**Private health insurance**	0.006	−0.09	0.10	0.89
**Care recipient length of kidney disease**	−0.08	−0.26	0.10	0.37
**Length of care**	0.07	−0.05	0.19	0.23
**Relationship type**	-0.0006	−0.12	0.12	0.99

The reference category for the difference are as follow: Age (every unit increase), Gender (male - female), Care recipient treatment (conservative care - dialysis), Country (UK-Australia), Education (completed high school or tertiary education - attended some high school or lower levels), Private health insurance (Yes - No/Unknown), Care recipient length of kidney disease (*≤*2 years - > 2 years), Length of care (0–2 years - > 2 years), Relationship type (Spouse/Partner – Child, sibling and other). SF-6D - Short Form six dimensions. CI - Confidence interval

### Care-related quality of life (CES scores)

Of 63 caregivers, the mean CES score for 61 with complete data was 74.41 (SD 17.67). The mean CES score of caregivers for patients managed with dialysis was 64.39 (16.75), and 80.91 (SD 15.20) for those caring for comprehensive conservative care patients (Additional file Table 1). The CES domain, “Getting on with the person you care for” reported the highest average score and was responsible for the greatest increment in overall CES score (Additional file Table 2).

In the univariate analyses, the mean CES score was 16.53 points lower for caregivers of dialysis patients than for comprehensive conservative care patients (*p* = 0.0002); and was 18.76 points lower for caregivers residing in the UK compared with Australia (*p* = 0.0003) (Additional file Table 1). Significant lower mean CES scores were observed for the spouse/partner compared with children of care recipients (Additional file Table 3).

When adjusted for other variables, the mean CES score was 15.73 points lower for caregivers of patients on dialysis compared with caregivers of patients on comprehensive conservative care (*p* = 0.003) (Table 3); and was 16.19 points lower for caregivers in the UK compared with Australia (*p* = 0.004) (Table 3).

**Table 3:** Adjusted differences in CES score according to caregiver sociodemographic characteristics and care recipient treatment group

	Differences	95% Lower CI	95% Upper CI	***p*** value
**Age (Y)**	0.01	−0.47	0.49	0.96
**Gender**	−1.81	−11.23	7.61	0.70
**Care recipient treatment**	−15.73	−25.68	−5.78	0.003*
**Health System**	16.19	5.63	26.75	0.004*
**Education**	−1.67	−11.70	8.36	0.74
**Private health insurance**	7.45	−3.79	18.69	0.19
**Care recipient length of kidney disease**	−15.52	−35.73	4.70	0.13
**Length of care**	−3.98	−17.11	9.16	0.55
**Relationship type**	4.31	−8.22	16.83	0.49

The reference category for the difference are as follow: Age (every unit increase), Gender (male - female), Care recipient treatment (conservative care - dialysis), Country (UK-Australia), Education (completed high school or tertiary education - attended some high school or lower levels), Private health insurance (Yes - No/Unknown), Care recipient length of kidney disease (*≤*2 years - > 2 years), Length of care (0–2 years - > 2 years), Relationship type (Spouse/Partner – Child, sibling and other). CES - Carer Experience Scale. * *p* < 0.05, statistical significance. CI - Confidence interval

## Discussion

The findings of this study give an insight into health-related and care-related quality of life of informal caregivers of older people with ESKD managed with dialysis or conservative care. Our prospective cross-sectional study suggests a significantly lower care-related quality of life for informal caregivers of older people on dialysis compared with those on conservative care, and those residing in the UK compared with those residing in Australia. However, no significant difference in HRQoL of the caregivers by care recipient treatment type or for any caregiver sociodemographic characteristics was observed. Providing care has a complex range of effects on caregiver’s quality of life and HRQoL instruments were specifically developed for measuring the impact of health and medical interventions on a patient’s quality of life rather than their caregiver’s [29, 41]. The lack of statistical significance in the HRQoL estimates could be attributed to the instrument’s insensitivity in measuring the effects of interventions on caregiver’s quality of life.

The caregivers of dialysis patients reported a mean CES score that was significantly lower than those caring for conservative care patients. A higher CES score indicates a higher care-related quality of life. The population norms for the CES scores are not available yet. However, a study into the construct validity of the CES in a heterogeneous group of carers in the UK presented mean CES scores and by category including duration of caring (< 20 h or ≥ 20 h per week), recipient’s health (bad, good to fair), and intensity of caring (not intense, relatively intense, intense) [42]. When comparing our study derived CES scores, it can be seen that the mean scores of caregivers of dialysis patients were consistent with providing an ‘intense’ level of care; to care recipient’s in ‘bad’ health; and for a caring duration ≥20 h per week [42].

The caregivers of dialysis patients reported a slightly lower utility (− 0.08) compared with the conservative care group reflecting a potentially clinically meaningful important difference related to treatment; however, this difference was not statistically significant. Meaningful differences or the minimal important difference (MID) in utility-based HRQoL reported in 11 studies using the SF-6D utilities, ranged from 0.011 to 0.097, with a mean MID of 0.041 [43]. It is therefore likely our study did detect a meaningful difference. In addition, the caregivers of dialysis patients reported a significant difference of − 15.73 in the CES score compared with conservative care group; however, the MIDs for CES has not yet been published. The lower CES score for caregivers of dialysis patients compared with those for conservative care patients observed in our study likely indicates the lower care-related quality of life.

The vitality domain representing energy/fatigue was observed to be the key driver affecting the HRQoL of caregivers of both the patient groups. It was reported to be much worse in the caregivers of dialysis patients compared with caregivers of conservative care patients, suggesting caregivers experiencing severe tiredness/fatigue as a result of providing care. We also observed that caregivers of dialysis patients had worse mental health scores compared with the caregivers of conservative care patients indicating poorer mental health. We could not find any studies comparing these two treatment groups specifically, but found previous studies reporting greater impairment to mental health and vitality domains of HRQoL of caregivers of dialysis patients [12, 44, 45]. In the care-related quality of life, we observed that the domain of ‘assistance from organisations and government’ received lower scores in both treatment groups compared with other domains. These suggest that caregivers may benefit from extra support, especially where the patient has a low HRQoL. Since some people may be reluctant to identify themselves as caregivers, limiting their ability to access support, health and social care professionals can play a role by encouraging caregivers to seek support [46] and signposting relevant help. Overall, we observed that the caregivers of conservative care patients reported higher scores for other domains such as ‘activities outside caring’, ‘support from friends and family’, and ‘control over caring’.

This study should be interpreted in the context of several limitations. First, no data on the caregiver’s comorbidities were collected. Considering the mean age of caregivers in this study, it is likely they might have some health problems. However, due to unavailability of the data, it was difficult to understand whether the strain of providing informal care lead to reductions in HRQoL, or whether people with health problems who become informal caregivers, perceived their tasks as being more straining [47]. Second, some differences in carer quality of life may be a result of the recipient’s dialysis modality. Our study did not have sufficient numbers of patients on peritoneal dialysis and haemodialysis to facilitate a meaningful comparison. Third, the cross-sectional design of the study does not allow inferences about the causality of health losses due to caregiving. Further research of the HRQoL of informal caregivers should be undertaken in longitudinal and controlled trials. Fourth, we only recruited 63 caregivers and a specific power calculation was not performed as the sample size was determined by the requirements of the original ICECAP-O study [36]. Although there could be a statistically significant difference in the mean scores, the study may not have been able to capture it due to smaller sample size. However, recruiting informal caregivers to research is a known challenge [48, 49]. Fifth, the study identified the principal caregiver of a patient, however, caregiving may also be shared around many relatives and friends. In this case, considering outcomes for a sole caregiver for each patient may understate the degree of spillover effects a healthcare intervention may have [29]. Finally, we did not have information on patient/caregiver’s ethnicity, a factor known to impact caregivers’ satisfaction with caregiving for older family members.

## Conclusion

The findings of this study suggest a lower care-related quality of life and greater need for support among informal caregivers of older patients with ESKD managed with dialysis compared with comprehensive conservative care. It is important to consider the impact on caregivers’ quality of life when considering treatment choices for their care recipients. Furthermore, measuring care-related quality of life using the CES alongside generic HRQoL measures, has the potential to provide a more detailed profile of the quality of life impacts on caregivers of older people with ESKD.

## Supplementary information

**Additional file 1: Table S1.** SF-6D utility and CES score according to caregiver characteristics and care recipient treatment group. SF-6D utility and CES scores for caregiver sociodemographic characteristic and care recipient treatment type. Hypothesis testing using t-test employed. **Table S2.** Mean scores and weights of SF-6D and Carer Experience Scale (CES) according to care recipient treatment group. Mean scores and weights for different domains of SF-6D and CES scale provided according to care recipient treatment type. **Table S3.** Differences in SF-6D utility and CES score based on the type of relationship with the care recipient. ANOVA analysis for the type of relationship variable (three categories - Spouse/Partner, Child, Others) for differences in SF-6D utility and CES score.

**Additional file 2: Figure S1.** Caregivers/patients flowchart. Flowchart on the number of caregivers included

**Additional file 3.** Item 1. STROBE Statement: checklist of items that should be included in reports of observational studies. STROBE checklist for cross-sectional studies for adequate and complete reporting of the study.Item 2. SF-12: Questionnaire (converted to SF-6D utilities). Administered to the caregivers. Item 3. Caregiver Experience Scale. Administered to the caregivers. Item 4. Background questions: (Caregivers). Administered to the caregivers.

## Data Availability

The datasets generated and/or analysed during the current study are not publicly available but are available from the corresponding author on reasonable request.

## Abbreviations

ESKD: End stage kidney disease
HRQoL: Health related quality of life
QALY: Quality adjusted life year
CES: Carer Experience Scale
SF-6D: Short Form Six Dimensions
SF-12: Short Form Survey 12 item
MID: Minimal important difference
