# Supplementary material for: Quality of life among caregivers of people with end-stage kidney disease managed with dialysis or comprehensive conservative care
Source: BMC Nephrol. 2020 May 4;21:160. doi: 10.1186/s12882-020-01830-9 (PMC7199363; doi:10.1186/s12882-020-01830-9)
Supplement: Supplementary file 1 — Additional file 1: Table S1. SF-6D utility and CES score according to caregiver characteristics and care recipient treatment group. SF-6D utility and CES scores for caregiver sociodemographic characteristic and care recipient treatment type. Hypothesis testing using t-test employed. Table S2. Mean scores and weights of SF-6D and Carer Experience Scale (CES) according to care recipient treatment group. Mean scores and weights for different domains of SF-6D and CES scale provided according to care recipient treatment type. Table S3. Differences in SF-6D utility and CES score based on the type of relationship with the care recipient. ANOVA analysis for the type of relationship variable (three categories - Spouse/Partner, Child, Others) for differences in SF-6D utility and CES score. [file 12882_2020_1830_MOESM1_ESM.pdf]

Additional file table 1: SF-6D utility and CES score according to caregiver characteristics and care recipient treatment group

Characteristics	SF-6D (n = 58)†		CES (n = 61)††	
	Mean ± SD	P-value	Mean ± SD	P-value
**Age**		0.16		0.90
≤76 years	0.76 ± 0.14		74.68 ± 18.78	
>76 years	0.71 ± 0.12		74.09 ± 16.61	
**Gender**		0.38		0.27
Males	0.76 ± 0.15		78.61 ± 18.16	
Females	0.73 ± 0.12		72.92 ± 17.46	
**Care recipient treatment‡**		0.05*		0.0002*
Dialysis	0.70 ± 0.13		64.39 ± 16.75	
Conservative care	0.77 ± 0.12		80.91 ± 15.20	
**Country**		0.96		0.0003*
United Kingdom	0.74 ± 0.13		59.96 ± 15.60	
Australia	0.74 ± 0.13		78.72 ± 16.00	
**Education ‡‡**		0.53		0.51
Attended some high school or lower levels	0.74 ± 0.12		75.89 ± 18.47	
Completed high school or tertiary education	0.73 ± 0.14		72.89 ± 16.98	
**Private health insurance §**		0.53		0.80
Yes	0.72 ± 0.15		74.82 ± 14.89	
No/Unknown	0.74 ± 0.12		73.60 ± 18.75	
**Care recipient length of kidney disease §§**		0.65		0.73
0-2 years	0.71 ± 0.15		77.05 ± 7.55	
>2 years	0.74 ± 0.13		74.08 ± 18.59	
**Length of care**		0.21		0.42
0-2 years	0.69 ± 0.12		76.80 ± 7.85	
> 2 years	0.74 ± 0.14		73.73 ± 19.38	

† SF-6D - Short Form six dimensions. Different SF-6D algorithms were used to convert the SF-12 scores to preference based SF-6D utilities for the UK and Australian population. 5 observations were missing from SF-6D calculations out of N = 63. †† CES - Carer Experience Scale. 2 observations were missing from CES calculations out of N = 63. ‡ The dialysis group consists of Facility Hemodialysis, Home Hemodialysis and Peritoneal dialysis. ‡‡ Individual responses to Primary school/Some high school were merged to one category as "Attended some high school or lower levels" and the responses to GCSEs/Completed high school/Diploma/TAFE/ Completed A- levels/ University degree were merged to one category as "Completed high school or tertiary education". § Individual responses to "No" and "Unknown" were merged to one category as "No/Unknown". §§ Individual responses to less than a year and 1 - 2 years of the length of kidney disease were merged to one category as 0-2 years. ¶ The responses varied from less than a year to more than 20 years and were grouped in two categories 0-2 years and > 2 years. * $p < 0.05$, statistical significance, the significance is between the sub-groups. CI - Confidence interval.

Additional file table 2: Mean scores and weights of SF-6D and Carer Experience Scale (CES) according to care recipient treatment group

Instrument	Dialysis (n=24)*		Conservative Care (n=37)	
	Score	Weights	Score	Weights
	Mean (SD)	Mean (SD)	Mean (SD)	Mean (SD)
**SF-6D‡**				
Physical functioning	2.27 (2.11)	-0.008 (0.02)	1.73 (0.69)	-0.006 (0.02)
Role limitations	2.85 (1.80)	-0.04 (0.03)	1.72 (1.16)	-0.02 (0.03)
Social functioning	3.04 (2.18)	-0.05 (0.03)	2.46 (1.52)	-0.05 (0.03)
Pain	2.46 (1.75)	-0.02 (0.03)	2.05 (0.97)	-0.02 (0.03)
Mental health	3.19 (1.88)	-0.06 (0.03)	2.32 (1.45)	-0.04 (0.03)
Vitality	3.42 (1.98)	-0.08 (0.01)	2.76 (1.36)	-0.08 (0.01)
**CES‡‡**				
Activities outside caring	1.88 (0.85)	9.96 (9.02)	2.46 (0.65)	16.23 (5.52)
Support from family and friends	2.08 (0.58)	11.85 (5.13)	2.70 (0.52)	16.10 (3.72)
Assistance from organisations and the government	1.46 (0.66)	4.43 (5.95)	1.92 (0.76)	8.50 (6.43)
Fulfilment from caring	2.54 (0.59)	12.16 (3.74)	2.59 (0.55)	12.51 (3.34)
Control over caring	2.29 (0.81)	10.95 (3.63)	2.65 (0.54)	12.61 (1.95)
Getting on with the person you care for	2.79 (0.41)	15.04 (4.12)	2.78 (0.42)	14.96 (4.15)

*2 observations missing from SF-6D scores and weights calculation. ‡ SF-6D domain scores are weighted decrements. ‡‡ CES domain scores are weighted increments. SD - Standard deviation.

Additional file table 3: Differences in SF-6D utility and CES score based on the type of relationship with the care recipient

	SF-6D (n = 58)†	CES (n = 61)††
**Relationship comparison**	**Difference (95 % CI)**	**Difference (95 % CI)**
**Spouse/Partner - Child**	-0.13 (-0.25; 0.006)	-17.22 (-34.18; -0.25)*
**Spouse/Partner - Sibling**	0.16 (-0.19; 0.51)	4.10 (-42.16; 50.36)
**Spouse/Partner - Other**	0.005 (-0.14; 0.14)	2.62 (-14.34; 19.59)
**Child - Sibling**	0.28 (-0.08; 0.64)	21.32 (-26.80; 69.43)
**Child - Other**	0.13 (-0.04; 0.31)	19.84 (-1.68; 41.36)
**Sibling - Other**	-0.15 (-0.52; 0.21)	-1.48 (49.59; 46.64)

† SF-6D - Short Form six dimensions. Different SF-6D algorithms were used to convert the SF-12 scores to preference based SF-6D utilities for the UK and Australian population. 5 observations were missing from SF-6D calculations out of N = 63. †† CES - Caring Experience Scale. 2 observations were missing from CES calculations out of N = 63. * $p < 0.05$, statistical significance, the significance is between the sub-groups. CI - Confidence interval.
