# Supplementary material for: Quality of life among caregivers of people with end-stage kidney disease managed with dialysis or comprehensive conservative care
Source: BMC Nephrol. 2020 May 4;21:160. doi: 10.1186/s12882-020-01830-9 (PMC7199363; doi:10.1186/s12882-020-01830-9)
Supplement: Supplementary file 2 — Additional file 2: Figure S1. Caregivers/patients flowchart. Flowchart on the number of caregivers included [file 12882_2020_1830_MOESM2_ESM.pdf]

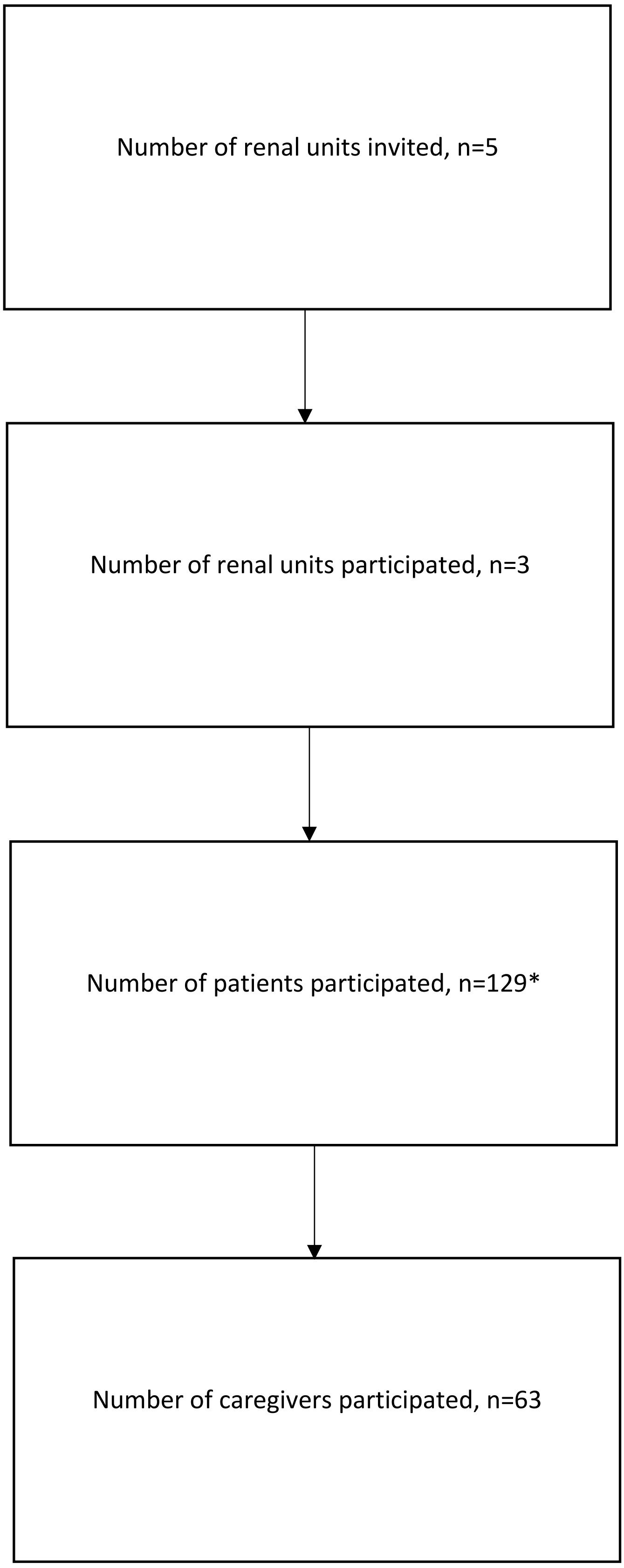

Additional file figure 1: Caregivers/patient flow chart

*Many patients did not have a caregiver (lived alone, spouse deceased) or caregivers did not consider participating in the study and declined.
