# Supplementary material for: Quality of life among caregivers of people with end-stage kidney disease managed with dialysis or comprehensive conservative care
Source: BMC Nephrol. 2020 May 4;21:160. doi: 10.1186/s12882-020-01830-9 (PMC7199363; doi:10.1186/s12882-020-01830-9)
Supplement: Supplementary file 3 — Additional file 3. Item 1. STROBE Statement: checklist of items that should be included in reports of observational studies. STROBE checklist for cross-sectional studies for adequate and complete reporting of the study.Item 2. SF-12: Questionnaire (converted to SF-6D utilities). Administered to the caregivers. Item 3. Caregiver Experience Scale. Administered to the caregivers. Item 4. Background questions: (Caregivers). Administered to the caregivers. [file 12882_2020_1830_MOESM3_ESM.pdf]

Additional file Item S1: STROBE Statement: checklist of items that should be included in reports of observational studies

	Item No	Recommendation	Yes/No/NA, Page No.
**Title and abstract**	1	(*a*) Indicate the study's design with a commonly used term in the title or the abstract	Yes, page 1-2
		(*b*) Provide in the abstract an informative and balanced summary of what was done and what was found	Yes, page 1-2
**Introduction**			
Background/rationale	2	Explain the scientific background and rationale for the investigation being reported	Yes, page 4-6
Objectives	3	State specific objectives, including any pre-specified hypotheses	Yes, page 5-6
**Methods**			
Study design	4	Present key elements of study design early in the paper	Yes, page 7
Setting	5	Describe the setting, locations, and relevant dates, including periods of recruitment, exposure, follow-up, and data collection	Yes, page 7
Participants	6	*Cross-sectional study*—Give the eligibility criteria, and the sources and methods of selection of participants	Yes, page 7

Variables	7	Clearly define all outcomes, exposures, predictors, potential confounders, and effect modifiers. Give diagnostic criteria, if applicable	Yes, page 8
Data sources/ measurement	8	For each variable of interest, give sources of data and details of methods of assessment (measurement). Describe comparability of assessment methods if there is more than one group	Yes, page 9
Bias	9	Describe any efforts to address potential sources of bias	NA
Study size	10	Explain how the study size was arrived at	Yes, page 9
Quantitative variables	11	Explain how quantitative variables were handled in the analyses. If applicable, describe which groupings were chosen and why	Yes, page 9
Statistical methods	12	(*a*) Describe all statistical methods, including those used to control for confounding	Yes page 9-10
		(*b*) Describe any methods used to examine subgroups and interactions	NA
		(*c*) Explain how missing data were addressed	Yes page 9-10
		Cross-sectional study—If applicable, describe analytical methods taking account of sampling strategy	Yes page 9-10
		(*e*) Describe any sensitivity analyses	NA

**Results**			
Participants	13*	(a) Report numbers of individuals at each stage of study—e.g., numbers potentially eligible, examined for eligibility, confirmed eligible, included in the study, completing follow-up, and analysed	Yes, page 10
		(b) Give reasons for non-participation at each stage	NA
		(c) Consider use of a flow diagram	NA
Descriptive data	14*	(a) Give characteristics of study participants (e.g., demographic, clinical, social) and information on exposures and potential confounders	Yes, page 10
		(b) Indicate number of participants with missing data for each variable of interest	NA
		(c) *Cohort study*—Summarise follow-up time (e.g., average and total amount)	NA
Outcome data	15*	*Cross-sectional study*—Report numbers of outcome events or summary measures	Yes, page 10-11
Main results	16	(*a*) Give unadjusted estimates and, if applicable, confounder-adjusted estimates and their precision (e.g., 95% confidence interval). Make clear which confounders were adjusted for and why they were included	Yes, page 10-11
		(*b*) Report category boundaries when continuous variables were categorised	Yes, page 10-11
		(*c*) If relevant, consider translating estimates of relative risk into absolute risk for a meaningful time period	NA

Other analyses	17	Report other analyses done—e.g., analyses of subgroups and interactions, and sensitivity analyses	NA
**Discussion**			
Key results	18	Summarise key results with reference to study objectives	Yes, page 12
Limitations	19	Discuss limitations of the study, taking into account sources of potential bias or imprecision. Discuss both direction and magnitude of any potential bias	Yes, Page 14-15
Interpretation	20	Give a cautious overall interpretation of results considering objectives, limitations, multiplicity of analyses, results from similar studies, and other relevant evidence	Yes, Page 13-15
Generalisability	21	Discuss the generalisability (external validity) of the study results	Yes, 13-15
**Other information**			
Funding	22	Give the source of funding and the role of the funders for the present study and, if applicable, for the original study on which the present article is based	Yes, page 16

Additional file Item S2: SF-12: Questionnaire (converted to SF-6D utilities)

Your Health

This survey includes a wide variety of questions about your health and your life. We are interested in how you feel about each of these issues.

1. In general, would you say your health is: [Mark an ☒ in the one box that best describes your answer.]

Excellent	Very good	Good	Fair	Poor
☐ 1	☐ 2	☐ 3	☐ 4	☐ 5

The following items are about activities you might do during a typical day. Does your health now limit you in these activities? If so, how much? [Mark an ☒ in a box on each line.]

	Yes, limited a lot	Yes, limited a little	No, not limited at all
**2.** Moderate activities, such as moving a table, pushing a vacuum cleaner, bowling, or playing golf	☐ 1	☐ 2	☐ 3
**3.** Climbing several flights of stairs	☐ 1	☐ 2	☐ 3

During the past 4 weeks, have you had any of the following problems with your work or other regular daily activities as a result of your physical health?

	Yes	No
**4.** Accomplished less than you would like	☐ 1	☐ 2
**5.** Were limited in the kind of work or other activities	☐ 1	☐ 2

During the past 4 weeks, have you had any of the following problems with your work or other regular daily activities as a result of any emotional problems (such as feeling depressed or anxious)?

	Yes	No
**6.** Accomplished less than you would like	☐ 1	☐ 2
**7.** Didn't do work or other activities as carefully as usual	☐ 1	☐ 2

8. During the past 4 weeks, how much did pain interfere with your normal work (including both work outside the home and housework)?

Not at all	A little bit	Moderately	Quite a bit	Extremely
☐ 1	☐ 2	☐ 3	☐ 4	☐ 5

These questions are about how you feel and how things have been with you <u>during the past 4 weeks</u>. For each question, please give the one answer that comes closest to the way you have been feeling.

How much of the time during the <u>past 4 weeks</u>…

	All of the time	Most of the time	A good bit of the time	Some of the time	A little of the time	None of the time
**9.** Have you felt calm and peaceful?	☐ 1	☐ 2	☐ 3	☐ 4	☐ 5	☐ 6
**10.** Did you have a lot of energy?	☐ 1	☐ 2	☐ 3	☐ 4	☐ 5	☐ 6
**11.** Have you felt downhearted and blue?	☐ 1	☐ 2	☐ 3	☐ 4	☐ 5	☐ 6

12. During the <u>past 4 weeks</u>, how much of the time has your <u>physical health or emotional problems</u> interfered with your social activities (like visiting with friends, relatives, etc.)?

All of the time	Most of the time	Some of the time	A little of the time	None of the time
☐ 1	☐ 2	☐ 3	☐ 4	☐ 5

Additional file Item S3: Caregiver Experience Scale

PLEASE TICK ONE BOX FOR EACH GROUP to indicate which statement best describes your current caring situation.

1. Activites outside caring (socialising, physical activity and spending time on hobbies, leisure or study)
You can do most of the other things you want to do outside caring
You can do some of the other things you want to do outside caring
You can do few of the other things you want to do outside caring

2. Support from family and friends (Personal help in caring and/or emotional support from family, friends, neighbours or work colleagues)
You get a lot of support from family and friends
You get some support from family and friends
Your get little support from family and friends

3. Assistance from organisations and the Government (Help fro public, private or voluntary groups in terms of benefits, respite and practical information)
You get a lot of assistance from organisations and the Government
You get some assistance from organisations and the Government
You get little assistance from organisations and the Government

4. Fulfilment from caring (Positive feeling from providing care, which may come from: making the person you care for happy, maintaining their dignity, being appreciated, fulfilling your responsibility, gaining new skills or contributing to the care of the person you look after)
You mostly find caring fulfilling
You sometimes find caring fulfilling
You rarely find caring fulfilling

5. Control over the caring (Your ability to influence the overall care of the person you look after)
You are in control of most aspects of the caring
You are in control of some aspects of the caring
You are in control of few aspects of the caring

6. Getting on with the person you care for (Being able to talk with the person you look after, and discuss things without arguing)
You mostly get on with the person you care for
You sometimes get on with the person you care for
You rarely get on with the person you care for

©Hareth Al-Janabi, Joanna Coast & Terry Flynn

Additional file Item S4: Background questions: (Caregivers)

Qu 1. What is your full name? ________________________________

Qu 2. What is your date of birth? _________________ (dd/mm/yyyy)

Qu 3. Gender (please tick one)
Male □ Female □

Qu 4. What is your main residential postcode? _________________

Qu 5. What was your country of birth? ________________________

Qu 6. What is the highest level of education you have completed? (please tick the box that best describes you)

Primary school	□
Some high school	□
Completed GCSEs	□
Completed A levels	□
Completed University Degree	□

Qu 7. Do you have private health insurance? (please tick one)

Yes	□
No	□
Don’t know	□

Qu 8. What type of kidney treatment is your family member/friend currently having? (please tick one)

Haemodialysis (satellite or hospital) □
Haemodialysis at home □
Peritoneal dialysis □
Non-dialysis renal supportive care □

Qu 9. How long have they had kidney disease? (please tick one)

Less than 12 months □
1-2 years □
More than 2 years □

Qu 10. How long have you been caring for this person?

__________________________ (months or years)

Qu 11. What is your relationship to this person? (please tick one)

Spouse/ partner □
Child □
Sibling □
Other □

Qu 12. The next 2 questions are about the SF-12 Survey. On the scale below please rate how easy this survey was to complete
(circle a number between 1 and 5)

Very easy	Somewhat easy	Neutral	Somewhat difficult	Very difficult
1	2	3	4	5

Qu 13. Did this survey measure the things that you consider important to your quality of life?
(circle a number between 1 and 5)

Completely agree	Somewhat agree	Neutral	Somewhat disagree	Completely disagree
1	2	3	4	5

Qu 14. If you responded with ‘somewhat disagree’ or ‘completely disagree,’ would you like to tell us what you think the SF-12 Survey was missing?

____
____
____
____
____

Qu 15. The next 2 questions are about the Carer Experience Scale. On the scale below please rate how easy this survey was to complete
(circle a number between 1 and 5)

Very easy	Somewhat easy	Neutral	Somewhat difficult	Very difficult
1	2	3	4	5

Qu 16. Did this survey measure the things that you consider important to your quality of life?
(circle a number between 1 and 5)

Completely agree	Somewhat agree	Neutral	Somewhat disagree	Completely disagree
1	2	3	4	5

Qu 17. If you responded with ‘somewhat disagree’ or ‘completely disagree,’ would you like to tell us what you think the Carer Experience Scale was missing?

____

____

____

____

Qu 18. Are there any other comments you would like to make about the surveys?

____

____

____

____

Thank you very much for taking the time to participate in this study. Please place your completed survey in the stamped envelope provided and hand back to the renal unit or post it to the university as soon as possible.
We greatly appreciate your assistance.
